# Photosynthetic markers reveal recovery of *Pisum sativum* from pathogen infection following fungicide treatment

**DOI:** 10.3389/fpls.2026.2005488

**Published:** 2026-10-30

**Authors:** Antonio J. Serrato, Paola Vargas, Aranzazu Peña, Sandy Fillet, Mariam Sahrawy

**Affiliations:** 1 Stress, Development and Signaling in Plants, Estación Experimental del Zaidín, Consejo Superior de Investigaciones Científicas, Granada, Spain; 2 Instituto Andaluz de Ciencias de la Tierra, Consejo Superior de Investigaciones Científicas, Granada, Spain; 3 Sanbiotec Consulting SL, Granada, Spain

**Keywords:** crop management, fungicide, markers, pathogen, pea, photosynthesis, photosynthetic proteins, pigments

## Abstract

Many plant-pathogen interactions can severely compromise plant growth and productivity, highlighting the need for effective strategies to prevent or mitigate infection and limit disease progression. Pathogen infection disrupts photosynthesis and carbon metabolism; however, the physiological consequences of fungicides, both beneficial and potentially phytotoxic, remain insufficiently characterised despite their widespread use in crop protection. After preliminary experiments in *Arabidopsis thaliana*, we investigated the impact of *Ascochyta pisi* infection on *Pisum sativum* and evaluated the efficacy of tebuconazole applied preventively or curatively. Determining the best time to apply the fungicide to the crops appears to be crucial. Photosynthetic function was assessed by PAM chlorophyll fluorometry, and pigment content and the abundance of selected chloroplastic proteins (cFBP1, TRX f, PsaC, D1/PsbA, and LHCII) were also determined. Infection caused a marked decline in Y(II) photosynthetic performance, accompanied by cFBP1 and TRX *f* decreased, and an induction of PsaC (PSI), the D1/PsbA protein (PSII), and LHCII. Curative tebuconazole application partially restored photosynthetic and metabolic functions, whereas preventive treatment did not fully counteract infection-induced alterations. Principal component analysis, integrating all measured variables, clearly separated the treatments and supported a stronger curative than preventive effect of the fungicide under the conditions tested. This combined physiological and biochemical approach enables robust comparisons across treatments and helps identify trade-offs between disease suppression and host performance. Overall, these results provide an integrative and quantitative framework using an effective and reliable methodology for assessing plant physiological responses to biotic stress and inform more sustainable crop protection strategies, particularly under increasing climate pressure in Mediterranean agroecosystems.

## 1 Introduction

Plant–pathogen interactions may induce significant physiological alterations in the host, notably affecting photosynthesis and carbon metabolism, which are central to energy production and plant development, respectively. Some pathogens target chloroplasts, compromising their structural integrity and function, thereby diminishing photochemical efficiency and can disrupt downstream metabolic pathways, such as those involved in the synthesis and degradation of carbohydrates, amino acid and lipids (Berger et al., 2007a, b; Rojas et al., 2014). Consequently, parameters such as, photosynthetic performance, pigment composition, photosystem protein abundance, and carbon metabolism serve as sensitive and integrative markers for detecting biotic stress and evaluating plant health before and after fungicide application (Pérez-Bueno et al., 2019).

Photosynthesis carries out the conversion of light energy into chemical energy in the form of adenosine triphosphate (ATP) and nicotinamide adenine dinucleotide phosphate (NADPH), primarily through the linear electron transport chain. Photosystems I (PSI) and II (PSII), the pigment-protein complexes of the light reactions, can be susceptible to oxidative stress induced by biotic agents such as pathogens. D1/PsbA protein is part of the core complex of PSII, and light-harvesting complex II (LHCII) is associated with PSII. While D1 is degraded, LCHII is mainly regulated, and photoinhibition (Baker, 2008; Campos et al., 2014). PSI components, including the PsaC protein subunit, are also affected, mainly after longer period of the stress. PSI is generally more resistant to photoinhibition than PSII, with PSII being the primary target of light-induced damage under most stress conditions (Sonoike, 2011; Powles, 1984). Photosynthetic pigments (chlorophylls and carotenoids) and anthocyanins, constitute critical indicators of plant physiological status because of their roles in light harvesting, photoprotection, and antioxidant defense; their relative abundance can reflect the plant capacity to withstand environmental and biotic stresses (Tamiaki and Kichishima, 2024; Li et al., 2025; Sun et al., 2022).

The Calvin-Benson cycle (CBC) utilises NADPH and ATP, generated during the light reactions, to fix atmospheric CO_2_ into carbohydrates. Key enzymes such as ribulose-1,5-bisphosphate carboxylase/oxygenase (Rubisco) and the chloroplastic and cytosolic fructose-1,6-bisphosphatase (cFBP1 and cyFBP, respectively), orchestrate the balance between sucrose and starch synthesis, optimising biomass accumulation (Rojas-González et al., 2015). Biotic stress frequently perturbs the synthesis and regulation of these enzymes, resulting in energy limitations and metabolic reprogramming that aims to restrict pathogen access to host resources (Kanwar and Jha, 2019; Scharte et al., 2005; Rojas et al., 2014). Monitoring the abundance of these carbon metabolism proteins provides insights into how the pathogen burden influences the assimilatory capacity and energy homeostasis of plants. Thioredoxin *f* (TRX *f*) is crucial for the redox activation of several CBC enzymes such as cFBP1, and contributes to the maintenance of chloroplast metabolic balance under changing environmental conditions (Serrato et al., 2004; Thormählen et al., 2015).

Fungicides used to mitigate pathogen-induced damage and restore photosynthetic function may, in some cases, exhibit phytotoxic effects, significantly influencing several physiological processes and thereby further compromising photosystem integrity and carbon metabolism (Petit et al., 2012). Hence, differentiating the physiological effects of pathogen infection from those attributable to fungicide treatments is critical for accurate agricultural assessments. However, the extent to which pathogens and fungicides affect the photosynthetic apparatus and primary metabolism remains insufficiently understood. It is therefore reasonable to implement a comprehensive monitoring of pathogen progression while evaluating the efficacy of preventive and curative fungicide applications. To address this, several methodologies have been developed to assess the performance of the photosynthetic apparatus. Among them, the non-invasive technique of pulse-amplitude-modulated (PAM) fluorometry has proven to be particularly effective for quantifying photosynthetic parameters, including the effective quantum yield of PSII [Y(II)], electron transport rate (ETR), and non-photochemical quenching (NPQ) (Lawson and Vialet-Chabrand, 2024). Information on photochemical efficiency, energy dissipation, and stress responses, facilitates real-time monitoring of plant health and treatment efficacy. All these parameters have been extensively studied under abiotic stress conditions (Liang et al., 2010), while their response to combined biotic and chemical stress remains less documented. Analysis of pigment composition and protein profiles of PSI, PSII, and of the CBC, provide valuable insights into the stability of the photosynthetic apparatus and the extent of stress-induced damage and facilitates the identification of reliable markers of plant physiological status, enabling the characterization of pathogen-induced responses, disease progression, and fungicide effects. This integrated approach supports comprehensive plant phenotyping and the optimisation of crop protection strategies (Lei, 2024).

A pertinent case study involves the legume *Pisum sativum* (*P. sativum*), an economically important legume crop, valued for its protein content and contribution to sustainable agriculture. Its cultivation is challenged by abiotic and biotic stresses, notably Ascochyta blight (anthracnose), caused by the fungal pathogen *Ascochyta pisi var pisi* (*A. pisi*) that affects pea crops in southern Spain. This disease severely affects photosynthetic capacity through leaf necrosis and tissue damage, thereby reducing light absorption, chlorophyll content, photosynthesis, and gas exchange, culminating in diminished plant vigour and yield with significant agronomic and economic repercussion (Annan et al., 2023). Disease management predominantly relies on fungicides such as tebuconazole (T); however, the optimal timing of application—whether applied preventively or after pathogen infection—remains unresolved. Therefore, understanding the interplay between *A. pisi* infection and photosynthetic performance, particularly under fungicide treatment, is essential for enhancing crop resilience and optimizing disease management strategies.

From a socioeconomic perspective, advancing knowledge of efficient and accessible management approaches is vital for improving agronomically important crops. Such strategies not only improve productivity but also bolster plant adaptive capacity in the face of escalating environmental stresses linked to global climate change, a concern particularly relevant in regions like Andalusia, where agricultural and environmental pressures are projected to intensify.

This study aims to integrate chlorophyll fluorescence imaging with physiological and proteomic assessments, and proposes a quantitative, comprehensive framework for monitoring disease progression and evaluating the efficacy of fungicide treatments.

## 2 Materials and methods

### 2.1 Plant material and growth conditions

*Arabidopsis thaliana.* Seeds of *Arabidopsis thaliana* (*A. thaliana*) wild type (WT) plants (ecotype Columbia-0, Col0) were initially used to determine the pesticide concentrations of interest and the general effects on the growth. Seeds were germinated and grown on Murashige and Skoog (MS) medium (Murashige and Skoog, 1962) for 20 days in a growth cabinet under long-day conditions (16 h light/8 h dark), with temperatures maintained at 22 °C during the light period and 20 °C during the dark period, and 60% relative humidity. The light intensity was set at 120 μmol m^-2^ s^-1^ photosynthetically active radiation (PAR).

*Pisum sativum.* Seeds of *P. sativum* were soaked in water for several hours prior to germination and then sown in a mixture of vermiculite/compost in a 1:3 (v/v) ratio. Plants were grown for 20 days in a growth chamber under long-day conditions at 22°C, with a 16h light photoperiod of 180 µmol m^-2^ s^-1^ PAR (Barajas-López et al., 2012). Plants were watered every two days. Three weeks after germination, leaves were treated with either water (C, control), the pathogen *A. pisi* (P) at one or different dilution levels, the fungicide tebuconazole (T), or a combination after 24h of both P and T or T and P.

### 2.2 Chemicals

Tebuconazole ((*RS*)-1-*p*-chlorophenyl-4,4-dimethyl-3-(1*H* - 1,2,4-triazol-1-ylmethyl) pentan-3-ol) is a systemic fungicide that is rapidly absorbed by vegetative plant tissues and translocated acropetally (Tomlin, 2003). It acts as a demethylation inhibitor (DMI) that interferes with ergosterol synthesis, a key component of the fungal cell membrane. This active ingredient (a.i.) is currently authorised for use in Spain (Ministerio de Agricultura, Pesca y Alimentación, 2022) and has been approved for the control of fungal diseases in crops such as peas and soybeans in Andalusia (southern Spain) (Perea Torres et al., 2015). A pesticide standard (Merck, purity ≥ 98.0%) was used without further purification. Tebuconazole stock solutions were prepared in dimethyl sulfoxide (DMSO) because of its low water solubility, and subsequent dilutions were made with sterile distilled water. The final DMSO concentration was maintained below 1% (v/v), which was not expected to affect seed germination or development.

### 2.3 *Arabidopsis thaliana* growth in MS containing tebuconazole

Based on the studies of Serra et al. (2013, 2019) in *A. thaliana*, a broad range of tebuconazole concentrations (between 0.625 and 20 mg L^-1^) has been previously evaluated. Accordingly, sterilised seeds of *Arabidopsis* WT plants were placed in four compartments Petri dishes (90 mm/4) and grown in 0.5x MS medium (Murashige and Skoog, 1962), with or without 0.5% sucrose (w/v), supplemented with 0 (T0), 0.3125 (T1), 0.625 (T2) and 2.5 (T3) mg L^-1^ tebuconazole in the respective compartments.

### 2.4 Determination of photosynthetic pigment and anthocyanins content

After 20 days, five *Arabidopsis* plantlets per different treatments were collected from Petri dishes and were weighed separately, and photosynthetic pigments extracted using 1% chlorhydric acid in methanol organic solvent. The contents of chlorophyll *a* (Chl *a*) and *b* (Chl *b*) (absorbance [Abs] at 665 nm and 652 nm, respectively), carotenoids (Abs 470 nm), and anthocyanins (Abs 530 nm and 657 nm) were quantified spectrophotometrically using a multiplate reader (Varioskan, Clariostar). The total chlorophyll content and Chl *a*/Chl *b* ratio were calculated according to established equations (Porra et al., 1989; Lichtenthaler and Wellburn, 1983; Lichtenthaler and Buschmann, 2001).

### 2.5 Determination of photosynthetic protein by western blot analysis

For protein extraction, a pooled sample of at least six plants (*Arabidopsis* rosette or pea leaves) was homogenized at a 1:3 (w/v) in an extraction buffer containing 25 mM Tris-HCl (pH 7.5), 5 mM MgCl_2_, 0.5 mM EDTA, 1mM PMSF, and 50% (v/v) glycerol. After centrifugation at 10,000 x g for 30 min at 4°C, protein concentration was determined using the Bradford method (Bradford, 1976) with a Bio-Rad protein assay kit. Western blotting was performed as described by Serrato et al. (2018) using specific antibodies. The antibodies used were anti-cFBP1, anti-TRX *f* generated in the laboratory; while the commercial´s were anti-PsaC (AS10 939), anti-PsbA/D1 (AS05 084), anti-LHCII (AS13 2705) and anti-actin (AS13 2640) (Agrisera AB, Vännäs, Sweden).

### 2.6 Pathogen infection and fungicide treatment

#### 2.6.1 Pathogen preparation and application

Pea plants were inoculated with the ascomycete *Ascochyta pisi*. The spore concentrations were preliminarily evaluated to determine the appropriate inoculum density. The *A. pisi* pathogen (P) was seeded in several Petri dishes with Potato Dextrose Agar (PDA) medium and incubated until significant fungal growth and sporulation were achieved. Spores were collected from the PDA plates and washed with sterile distilled water. The resulting spore suspension was quantified using a Neubauer counting chamber, yielding a mean concentration of 2 x 10^4^ spores/ml. Serial dilutions of 1/10, 1/100, 1/1,000, and 1/10,000 were prepared using sterile water. Three-week-old pea leaves were then inoculated with two to three sprays of either undiluted or diluted spore suspensions, or sterile water as a control. After 5 days, leaves were collected and phenotypic symptoms were assessed (**Figure 1**) in relation to disease severity. Protein extraction was subsequently performed, and western blot analyses were performed using anti-TRX *f* (thioredoxin *f*) as a stress redox marker response (**Supplementary Figure 1**).

**Figure 1:**
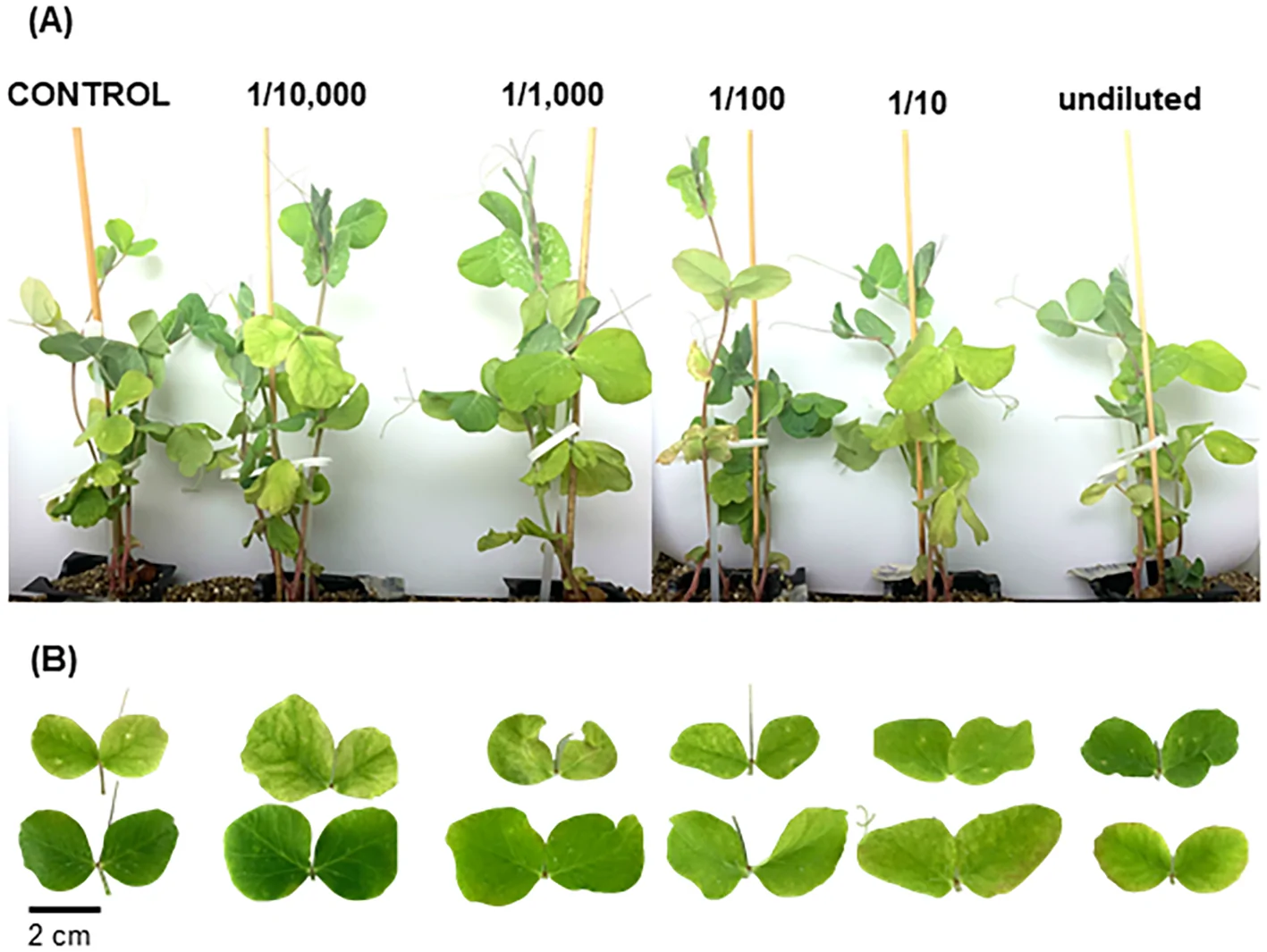
*Pisum sativum* plants infected with *Ascochyta pisi* at different inoculum dilutions (undiluted, 1/10, 1/100, 1/1,000, 1/10,000) and control. **(A)** Representative pea plantlets. **(B)** Close-up view of infected leaves showing disease symptoms and control plant leaves.

#### 2.6.2 Experimental design of pathogen infection and fungicide treatment on pea plants

Three-week-old *P. sativum* plantlets were used in this experiment. Four treatments were applied at different periods and in different combinations: Control (C), Pathogen (P), Tebuconazole + Pathogen (TP) and Pathogen + Tebuconazole (PT). Each plant received five sprays per plant leaf and a minimum of four to five plant replicates per treatment were performed. On the first day (D0), control plants (C) were sprayed with water. Plants of the treatment P group were inoculated with *A. pisi* at a 1/1,000 dilution prepared by diluting 20 µL in 20 ml sterile distilled water. Finally, T plants group were sprayed with the fungicide tebuconazole at 2.25 mg L^-1^ supplemented with 0.05% Silwet L-77. After 24 h (day 1, D1), plants from treatments C, P and T were analysed using Fluor PAM. Subsequently, half of the plant leaves from treatment P were sprayed with tebuconazole and reassigned as PT. Conversely, plant leaves from treatment T were inoculated with *A. pisi* and reassigned as TP. Imaging PAM analyses of treatments C, P, PT and TP were repeated at day 2 (D2, 48 h from the initial treatment and 24 h after the second application) and day 9 (D9; 9 days after the initiation of the experiment). Finally, on Day 9 (D9) pea plant leaves were collected for subsequent quantification of pigment, and frozen in liquid N_2_ and stored at -80 °C for metabolic protein analysis, following the methods described in chapter 2.4 and 2.5.

### 2.7 PSII photochemical efficiency and photoprotective parameters determination

Chlorophyll fluorescence measurements and imaging were performed using an imaging pulse-amplitude-modulated fluorometer (IMAGING PAM system; Heinz Walz, Effeltrich, Germany; www.walz.com). The instrument uses an array of blue light-emitting diodes (LEDs; peak wavelength 470 nm) for fluorescence excitation, actinic illumination, and the application of saturating light pulses. Prior to measurements, the pea plants were dark-adapted for 30 minutes. Fully expanded young pea leaves were selected for analysis, and measurements were conducted across the five treatments (C, T, TP, P and PT) as illustrated in **Figure 2A**. The maximum quantum yield of PSII (F_v_/F_m_) was calculated as F_v_/F_m_=(F_m_-F_0_)/F_m_, where F_0_ represents the initial minimal fluorescence emitted from dark-adapted leaves, and F_m_ corresponds to the maximal fluorescence induced by a saturating actinic light pulse. Variable fluorescence (F_v_) is defined as the difference between F_m_ and F_0_, reflecting the variable component of chlorophyll fluorescence associated with the photochemical capacity of PSII. A series of actinic light steps ranging from 0 to 1,251 µmol m^−2^ s^−1^ photosynthetic photon flux density (PPFD) were applied for 30 s at each irradiation level to determine the photosynthetic electron transport rate (ETR) of the leaves. Saturation pulses (2,700 µE m^-2^ s^-1^ for 0.8 s) were applied to determine maximal fluorescence in both dark- and light-adapted states (F_m_ and F_m’_, respectively). The effective quantum yield of PSII (Y(II)), which represents the efficiency with which absorbed light is used for photochemistry in PSII under light-adapted conditions, was calculated as Y(II) = (F_m’_-F’)/F_m’_. The fraction of open PSII reaction centres qL was calculated as qL = (F_m’_-F)/(F_m’_-F_0’_) x F_0’_/F. Non-photochemical quenching (NPQ), which reflects photoprotective energy dissipation as heat, was calculated as NPQ = (F_m_-F_m’_)/F_m’_, where the value of Fm was measured after relaxation of NPQ following 30 minutes of dark adaptation (Li et al., 2002; Fu et al., 2012). Additionally, measurements were made of the quantum yield of regulated non-photochemical energy dissipation Y(NPQ), which represents the photoprotective component of energy loss via controlled heat dissipation mechanisms, and the quantum yield of non-regulated non-photochemical energy dissipation Y(NO) reflects constitutive, non-regulated energy losses associated with photoinhibition and other uncontrolled processes.

**Figure 2:**
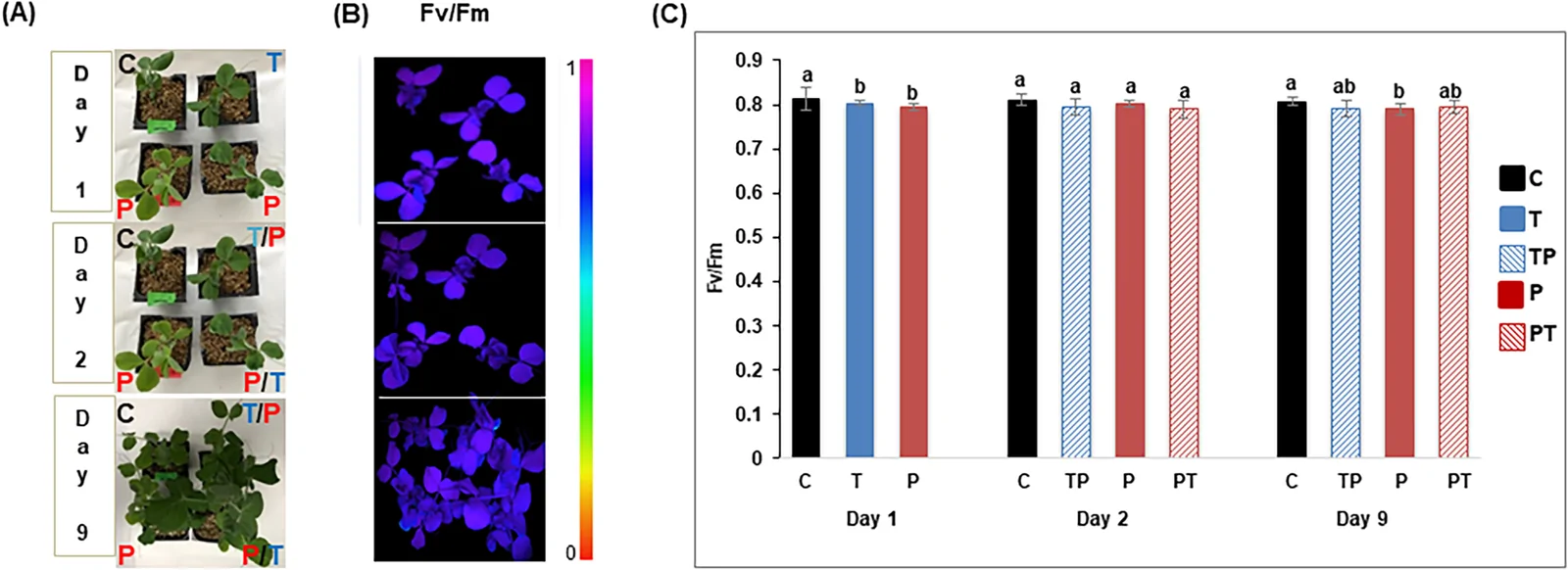
Chlorophyll fluorescence analysis of *P. sativum* plants subjected to tebuconazole (T) or *A. pisi* (P) infection in comparison to control conditions (C). Assessments were performed on Day 1 (D1, 24h after treatment) and additional treatments were performed, T leaves plants were inoculated with *A. pisi* (TP; tebuconazole-treated leaves subsequently inoculated with the pathogen) and P leaves plants were sprayed with T (PT; pathogen-inoculated leaves subsequently sprayed with tebuconazole). On Day 2 (D2, 48h after treatment), new assessments were performed as well as on Day 9, after 9 days the initiation of treatments. **(A)** Representative plant phenotypes and distribution. **(B)** Visualization of the maximum quantum yield of photosystem II (F_v_/F_m_) obtained using an imaging PAM fluorometer at D1, D2 and D9. **(C)** F_v_/F_m_ values. Data presented as the mean ± standard error (SE). Different letters mean statistically significant differences among treatments according to a one-way analysis of variance (ANOVA) followed by Tukey’s *post hoc* test (p<0.05). Three biological replicates were analysed, and F_v_/F_m_ values were determined from nine fully expanded leaves from three plants per treatment.

### 2.8 Statistical analysis

Statistical analysis was performed using analysis of variance (ANOVA) to assess significant differences among treatments. Data normality was tested using the Shapiro–Wilk test, and homogeneity of variances was established using Levene’s test (p>0.05). One-way ANOVA was performed, followed by Tukey´s HSD test. When data were non-normally distributed, non-parametric analysis was performed using Kruskal–Wallis test, followed by Dunn’s test. Principal Component Analysis (PCA) was performed using R software (RStudio; https://rstudio.com), and mean comparisons were carried out using Tukey´s HSD test implemented in the “agricolae package”, available online at https://CRAN.R-project.org/package=agricolae). All statistical analyses were conducted at a significance level of p ≤ 0.05.

## 3 Results

### 3.1 *Arabidopsis thaliana* growth response to tebuconazole

The range of the fungicide tebuconazole concentrations used in the preliminary experiments with *A. thaliana* seeds was based on recommended agricultural doses for different plant species and on data from the literature (Serra et al., 2013, 2019). As shown in **Figure 3**, the dose-response analysis using increasing tebuconazole concentrations in the growth medium (T0 to T3) affected *Arabidopsis* seedling development, resulting in a general reduced seedling size, mainly at the T3 dose. However, sucrose demonstrated a protective effect against fungicide toxicity (**Figure 3A**). *Arabidopsis* plantlets grown on sucrose-supplemented medium (T+) exhibited a visibly healthier phenotype, with similar biomass (**Figure 3B**), compared to those cultivated without sucrose (T-). In the absence of sucrose, a marked growth reduction was observed, particularly at 2.5 mg L^-1^, where plant growth and biomass were reduced by at least 75% (**Figures 3A, B**). The cFBP1 content, as determined by western blotting, decreased with increasing fungicide concentration (**Figure 3C**). This decline in cFBP1 was more pronounced in *Arabidopsis* grown in sucrose-deficient medium.

**Figure 3:**
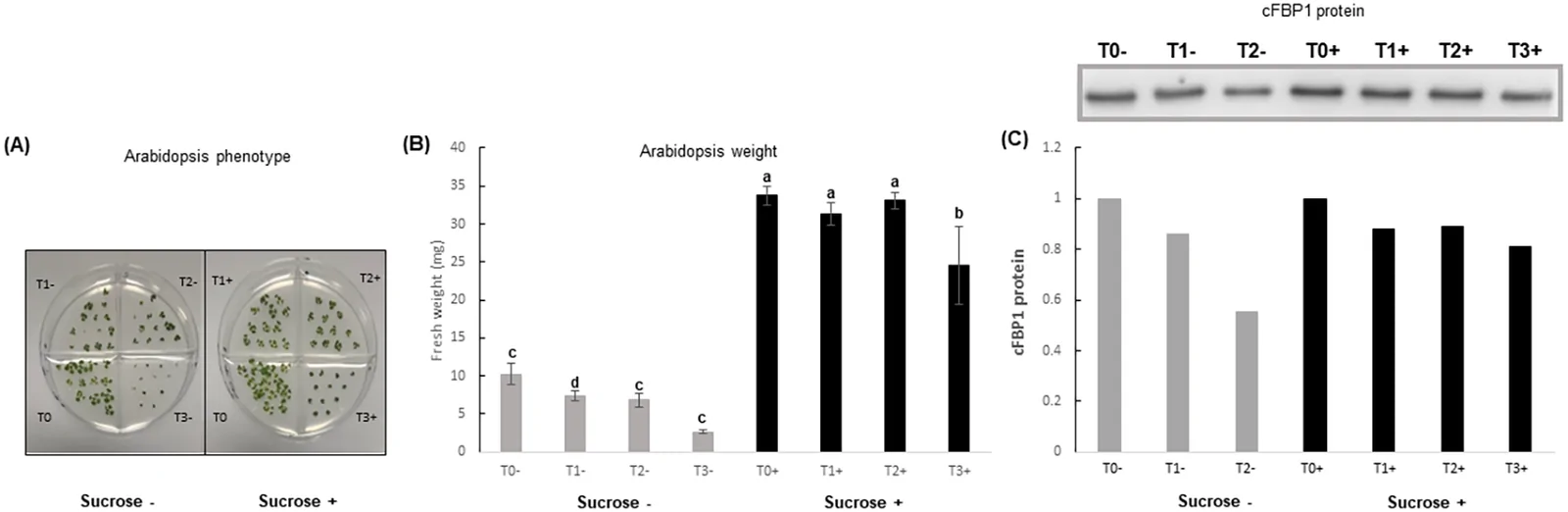
**(A)** *Arabidopsis thaliana* seeds grown in microtiter plates containing MS medium without (T-) or with (T+) sucrose and supplemented with either no fungicide (T0, control) or three concentrations of the systemic fungicide Tebuconazole (T, T1 0.3125 mg L^-1^; T2 0.625 mg L^-1^; T3 2.5 mg L^-1^). **(B)** Fresh weight of Arabidopsis plantlets grown under the same conditions as described in **(A)**. **(C)** Western blot analysis of cFBP1 (chloroplastic fructose-1,6-bisphosphatase) and quantification of protein abundance.

### 3.2 Pigment content in *Arabidopsis* plants treated with tebuconazole

**Table 1** shows the content of plant pigments chlorophyll *a* and *b*, carotenoids and anthocyanins, determined in *Arabidopsis* grown on MS medium containing increasing concentrations of the fungicide (T0 to T3) either with (T+) or without (T-) sucrose. When *A. thaliana* seedlings were grown in a medium supplemented with sucrose, the highest fungicide concentration of 2.5 mg L^-1^ resulted in significant differences in chlorophyll *a* and *b*, carotenoid, and anthocyanin contents, whereas lower concentrations, including the control, showed no statistically significant differences. The chlorophyll a/b ratio, an indicator of photosynthetic apparatus efficiency, remained unchanged across the range of concentrations tested. In the absence of sucrose (T-), the two highest concentrations (0.625 and 2.5 mg L^-1^ tebuconazole) differed significantly from the lower concentrations and resulted in higher pigment content than that in plants grown in sucrose containing medium. Anthocyanin and carotenoid contents (T2- and T3-) in *A. thaliana* grown without sucrose increased markedly compared to those grown with sucrose. This indicated a well-defined defense response to stress generated by the fungicide, characterised by the accumulation of carotenoids and anthocyanins (**Table 1**; **Figure 3A**).

**Table 1:** Plant pigment content (mg L^-1^) of *A. thaliana* grown in medium with different tebuconazole concentrations and with (T+) or without (T) sucrose.

Pesticide concentration (mg L^-1^)	Chl *a*	Chl *b*	Chl *a*+*b*	Ratio Chl *a*/Chl *b*	Carotenoids	Anthocyanin
Tebuconazole						
T0+ (0)	0.513 ± 0.129 a	0.336 ± 0.079 a	0.848 ± 0.032 a	1.535 a	0.084 ± 0.032 a	0.0015 ± 0.0007 a
T1+ (0.3125)	0.493 ± 0.276 a	0.273 ± 0.150 a	0.719 ± 0.377 a	1.687 a	0.064 ± 0.017 a	0.0021 ± 0.0009 a
T2+ (0.625)	0.382 ± 0.048 a	0.254 ± 0.077 a	0.636 ± 0.110 a	1.369 a	0.036 ± 0.011 a	0.0010 ± 0.0002 a
T3+ (2.5)	1.077 ± 0.190 b	0.646 ± 0.064 b	1.629 ± 0.405 b	1.802 a	0.196 ± 0.030 b	0.0065 ± 0.0020 b
T0- (0)	1.140 ± 0.117 a	0.722 ± 0.071 a	1.658 ± 0.465 a	1.594 ab	0.224 ± 0.046 a	0.0040 ± 0.0010 a
T1- (0.3125)	1.408 ± 0.162 a	0.835 ± 0.284 a	2.243 ± 0.438 a	1.795 ab	0.254 ± 0.079 a	0.0057 ± 0.0009 a
T2- (0.625)	5.041 ± 0.318 b	2.697 ± 0.606 b	7.738 ± 2.282 b	2.050 b	1.226 ± 0.297 b	0.0305 ± 0.0109 b
T3- (2.5)	7.275 ± 0.797 c	6.111 ± 1.407 c	13.386 ± 1.989 c	1.220 a	0.905 ± 0.447 b	0.0544 ± 0.0173 c

Different letters in the same column for the same treatment (with or without) indicate significant differences (p ≤ 0.05) (n=5).

### 3.3 Plant, pathogen and fungicide interactions

#### 3.3.1 Pathogen and fungicide settings

Foliar assessment of *P. sativum* inoculated with *A. pisi* showed a clear dose-dependent development of disease symptoms (**Figure 1A**). The control leaves and those inoculated with highly diluted suspensions (1/10,000 and 1/1,000, respectively) were visually asymptomatic and apparently uniformly green, but biochemically detectable when TRX *f* was determined as it is described below. Mild chlorosis and initial leaflet deformation were first observed at a 1/100 dilution. In contrast, higher inoculum densities (1/10 and undiluted suspension) caused severe symptoms, including chlorosis, few necrotic lesions, and chlorotic marginal wrinkling, leading to a reduction and distortion of leaflet area (**Figure 1B**). Based on these results, a 1/1,000 dilution was selected as the optimal inoculum concentration for *A. pisi* infection (P) to induce a mild yet reproducible stress response in pea leaves, at which the leaf phenotypes were at the threshold between phytotoxicity and resistance without causing severe damage. This inoculum concentration aligns with the low to moderate natural infection pressure described for foliar pathogens in legume crops under field conditions (Jiménez-Vaquero and Rubiales, 2026). The selected dilution enabled sustained physiological monitoring and reliable photosynthetic measurements throughout the experimental period while still eliciting a clearly detectable stress response. At this inoculum concentration (1/1,000), and after five days, TRX *f* redox marker, assessed by western blotting, reached the average level, indicating an intermediate oxidative stress response triggered by the infection (**Supplementary Figure 1**).

For our experimental design, we used tebuconazole as the selected fungicide to control *A. pisi* at the concentration recommended in the literature for pea crop. As described in the Materials and Methods section, a combination of treatments was used to evaluate the role of tebuconazole in protecting pea plants from *A. pisi* infection before or after application. **Figure 2A** shows the representative distribution of pea plants subjected to different treatments, C (water), T (tebuconazole; 2.25 mg L^-1^) and P (pathogen *A. pisi*; Dil. 1/1,000). At D1 (C, P, T), D2 and D9 (C, P, PT, TP) chlorophyll fluorescence measurements were performed. **Figure 2B** shows the differences in the visualization of the maximum quantum yield of PSII by using imaging PAM fluorometer, and **Figure 2C** displays data of F_v_/F_m_.

#### 3.3.2 Chlorophyll fluorescence: PSII quantum yield and photoprotective mechanism

The analysis of chlorophyll fluorescence in dark-adapted pea plants using Imaging PAM could evaluate different parameters and the selected images of F_v_/F_m_, Y(II), Y(NPQ) and qL shown in **Figures 2** and **4** indicate relevant differences between treatments after D1, D2 and D9 and the timing of their application. The maximal quantum yield of PSII, F_v_/F_m_, indicates that both tebuconazole and *A. pisi* affect PSII efficiency and reflect a stress condition in pea plants (**Figures 2B, C**). In our study, F_v_/F_m_ values ranged from 0.79 to 0.83 on D1, from 0.76 to 0.83 on D2, and from 0.78 to 0.82 D9. Significant differences were observed after 24 h between C and T and P plants (p<0.001), whereas on D9 the differences between C and P were of p<0.05. These differences were slightly less marked between C and TP and PT treated plants on D9. In contrast, no significant differences were observed among the four treatments after 48 h (D2) (p>0.05). These results indicate that F_v_/F_*m*_ was affected by both the pathogen and fungicide. However, tebuconazole application, either before or after infection, appears to protect to some extent PSII efficiency.

**Figure 4:**
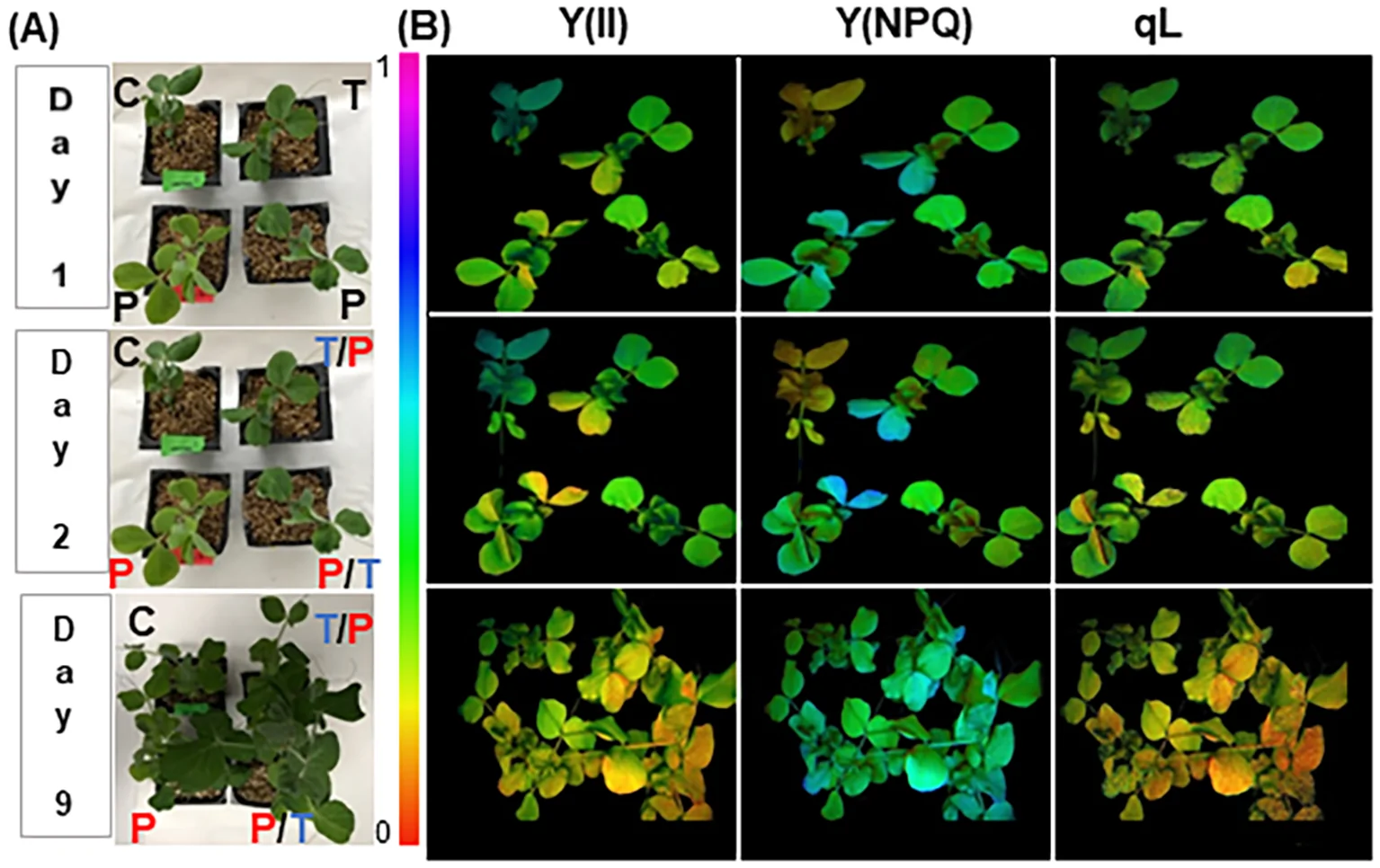
Imaging-PAM Imaging of photosynthetic performance (PSII) in *P. sativum* plants subjected to tebuconazole (T) or/and *A. pisi* (P) infection in comparison to control conditions (C). After Day 1 (D1, 24h hours treatment) the parameters analysed were the effective quantum yield of photosystem II [Y(II)], the quantum yield of regulated non-photochemical energy dissipation [Y(NPQ)], and the fraction of open PSII reaction centers (qL); and additional treatments were performed, T leaves plants were inoculated with *A. pisi* (TP; tebuconazole-treated leaves subsequently inoculated with the pathogen) and P leaves plants were sprayed with T (PT; pathogen-inoculated leaves subsequently sprayed with tebuconazole). On Day 2 (D2, 48h after treatment), and on Day 9 (D9 after 9 days the initiation of treatments) new assessments were performed. **(A)** Representative plant phenotypes and distribution. **(B)** Visualization of Y(II), Y(NPQ) and qL obtained using an imaging PAM fluorometer at D1, D2 and D9.

Concerning the fluorescence images corresponding to Y(II), Y(NPQ), and qL, it can be seen clear differences in response to *A. pisi* infection and tebuconazole application over the 9 days experiment (**Figure 4**). Leaves of P and TP treated plants display a more chlorotic and wrinkle aspect and smaller size in relation to C and PT. The qL parameter values (**Supplementary Table 1**), which describes the proportion of open PSII centers in leaves (Kramer et al., 2004) was very similar across all measurements, with only sporadic differences observed between the control and the other treatments on D2.

**Figure 5** shows the light response curves of ETR, Y(II) and NPQ on D1, D2 and D9 in pea plants subjected to the different treatments. Regarding the early plant responses on D1, ETR decreased significantly (by at least 50%) under tebuconazole or *A. pisi* action following the initial treatments. However, on D2, the PT treatment (tebuconazole applied on P plants) was the only treatment that did not differ from the control, whereas treatments P and TP continued to show values similar to those observed at D1. By D9, all values had decreased compared to D1 and D2, and all treatments affected the plants compared to control plants. In addition to the effect of treatment type and combination, these data confirm that light intensity plays an important role in plant response to *A. pisi* infection. Above 230 µmol photons m^-2^ s^-1^, light curves began to diverge. In contrast, at D9, 111 µmol photons m^-2^ s^-1^ was the light intensity at which the differences among treatments became evident. A similar trend was observed for Y(II). A marked decrease in Y(II) was detected at D1 under T and P treatments and at D2 under P treatments. However, at D2, the reduction was less pronounced when T was applied (PT). This suggests that T may partially protect pea plants from infection. Nevertheless, by D9, Y(II) decreased drastically under all treatments compared to C. Differences between the control and treated plants in light-response curves were consistently observed at 146 µmol photons m^-2^ s^-1^ at all sampling times.

**Figure 5:**
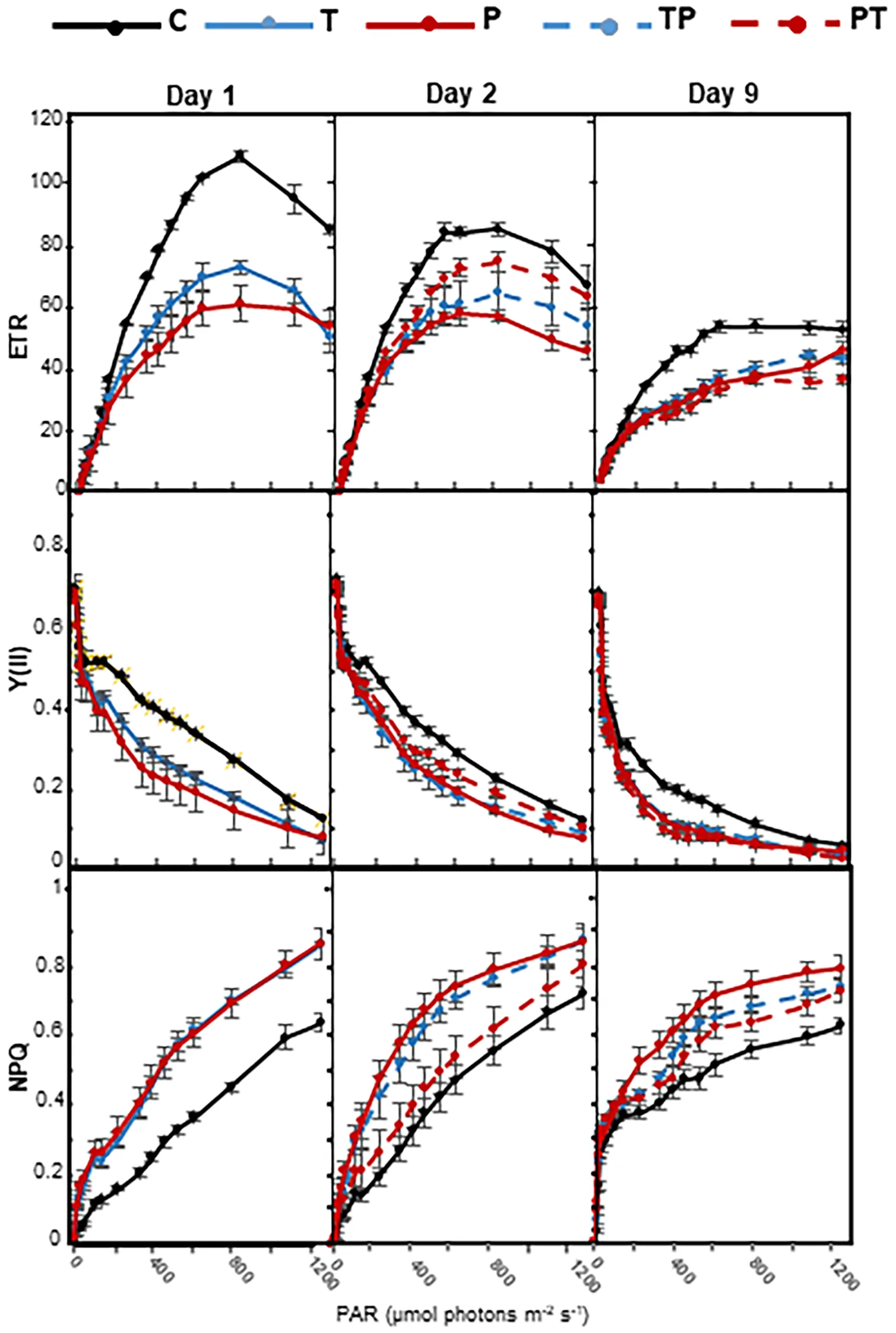
Chlorophyll Fluorescence analysis of the electron transport rate (ETR), effective quantum yield of photosystem II (Y(II), and non-photochemical quenching of dissipation of excess energy (NPQ) in *P. sativum* plants grown during 24h (Day 1) under control conditions (C), sprayed with Tebuconazole (T) or infected by *A. pisi* (P). Additional treatments were performed, T leaves plants were inoculated with *A. pisi* (TP; tebuconazole-treated leaves subsequently inoculated with the pathogen) and P leaves plants were sprayed with T (PT; pathogen-inoculated leaves subsequently sprayed with tebuconazole). On Day 2 (D2, 48h after treatment), and on Day 9 (D9 after 9 days the initiation of treatments) new analysis were performed.

At D1, NPQ which reflects the energy dissipation not used for photochemistry (Ritchie and Bunthawin, 2010), showed a significant increase (~20%) in plants subjected to P and T treatments compared with C. Interestingly, after 2 days (D2), no statistically significant differences in NPQ were observed between the control and PT treatment. These findings suggest that the application of T to infected plants exerts a transient curative effect avoiding increasing stress. On D9, P infection alone maintained the stress effect, whereas the combined treatments (PT and TP) appeared to mitigate the effect of *A. pisi* infection. By the end of the experiment, the plant defense system had further evolved, and in general, all measured photosynthetic parameters in the control differed significantly from those of treated plants.

Total non-photochemical energy dissipation can be partitioned into regulated non-photochemical quenching energy dissipation [Y(NPQ)] and non-regulated non-photochemical dissipation [Y(NO)], the latter representing the fraction of energy passively dissipated as heat and fluorescence (Fu et al., 2012) associated with photodamage and PSII dysfunction (Kramer et al., 2004; Klughammer and Schreiber, 2008). In this study, as shown in **Figure 6**, both Y(NPQ) and Y(NO) values reflected distinct stress responses in pea plants subjected to P infection and T treatment. At D1 and D2, Y(NPQ) exhibited a pattern similar to that of the NPQ light response curves (**Figure 5**). At D2, PT plants showed Y(NPQ) values approximately 25% closer to those of the control and lower than those of plants subjected to P or TP treatments (**Figure 6**). By D9, significant differences in Y(NPQ) were evident between control and treated plants. P, PT and TP treated plants displayed similar Y(NPQ) values, all higher than those of the control plants, indicating enhanced regulation of Y(NPQ) under these conditions. Interestingly, at D1, Y(NO) of C plants differed in a light dependent manner from that of P or T leaves. At an irradiance starting at 336 µmol photons m^-2^ s^-1^ and above, C plants exhibited the highest Y(NO) values. At this same irradiance, the P plants light-response curve began to decline and continued to decrease progressively up to the maximum irradiance (1251 µmol photons m^-2^ s^-1^), following a pattern similar to that of the T treatment light curve. Plants T treated consistently exhibited the lowest Y(NO) values across the entire irradiance range. A comparable pattern was observed at D2 for Y(NO) in relation to Y(NPQ) and NPQ. These results indicate that, at both D1 and D2, energy dissipation induced by T or P application occurred predominantly via a regulated non-photochemical mechanism. On D9, only PT plants exhibited significantly higher Y(NO) values than the control plants. Notably, these differences were restricted to intermediate light intensities (150-450 µmol photons m^-2^ s^-1^), whereas no significant differences were observed at higher irradiances under all treatments. At this stage, energy dissipation involves a greater contribution from non-regulated non-photochemical processes. Overall, these results suggest that T application moderately enhances PSII photoprotection in pea *A. pisi* infected plants through energy dissipation.

**Figure 6:**
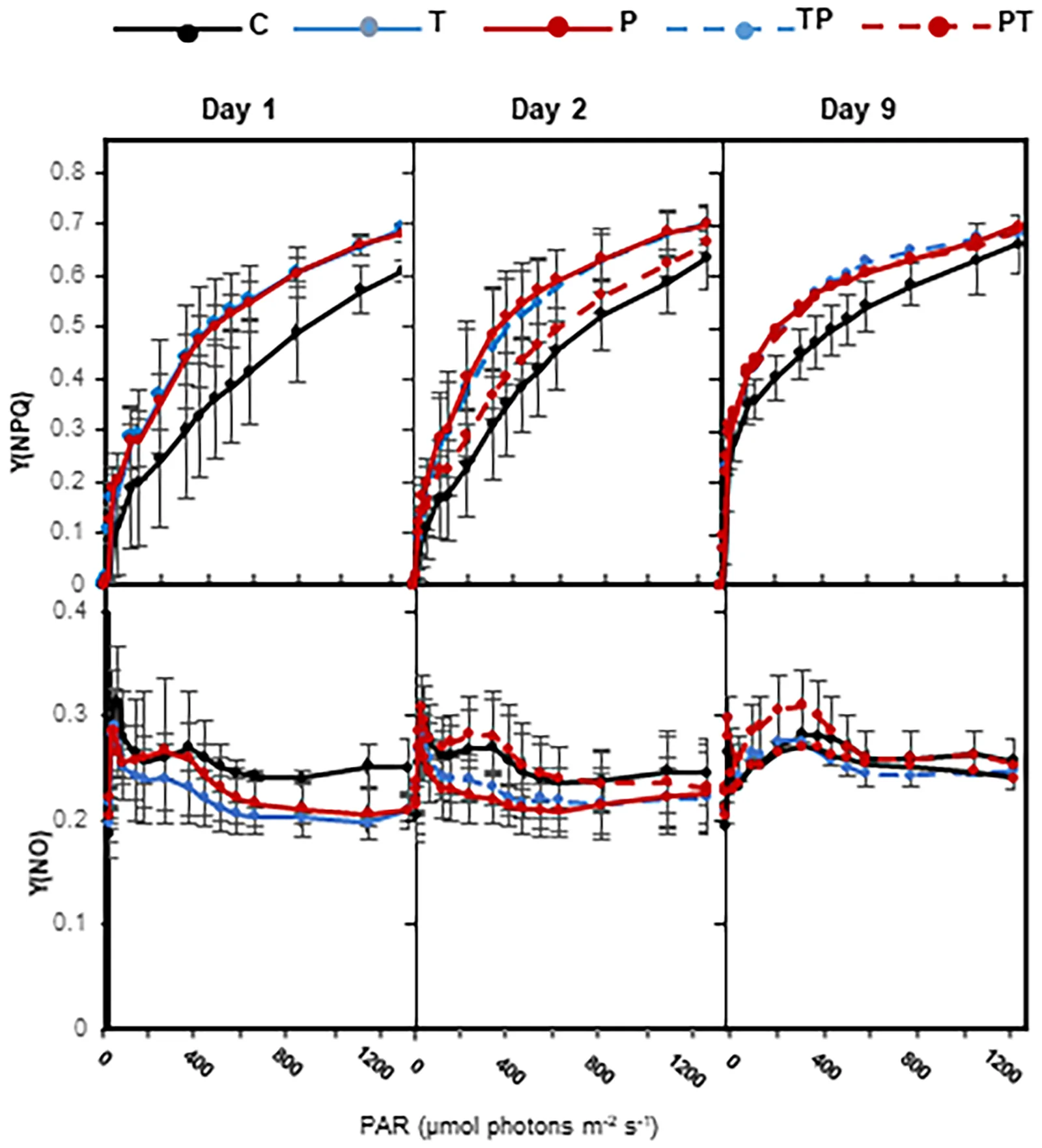
Chlorophyll Fluorescence analysis of values for quantum yield of regulated non-photochemical energy loss (Y(NPQ)) and quantum yield of non-regulated and non-photochemical quenching (Y(NO)) in *P. sativum* plants grown during 24h (Day 1) under control conditions (C), sprayed with tebuconazole (T) or infected by *A. pisi* (P). Additional treatments were performed, T leaves plants were inoculated with *A. pisi* (TP; tebuconazole-treated leaves subsequently inoculated with the pathogen) and P leaves plants were sprayed with T (PT; pathogen-inoculated leaves subsequently sprayed with tebuconazole). On Day 2 (D2, 48h after treatment), and on Day 9 (D9 after 9 days the initiation of treatments) new analysis were performed.

#### 3.3.3 Pigment content

In plants subjected to TP and PT treatments, chlorophyll *a* concentration exhibited a slight increase relative to the control, while in P plants this pigment diminished (**Figure 7**). In contrast, chlorophyll *b* levels were slightly reduced across all treatments compared with the control. Although these differences were not statistically significant. This divergent response between the two major chlorophyll pigments resulted in a significantly higher Chl *a*/Chl *b* ratio in treated plants compared to the control. This Chl *a*/Chl *b* ratio is widely recognised as a sensitive indicator of adjustments in the light-harvesting complexes (LHCs) and the relative distribution of photosystem antenna pigments (Tanaka and Tanaka, 2007; Lokstein et al., 2021). In contrast, carotenoid and anthocyanin content, two pigment groups associated with photoprotection, antioxidant defense, and stress tolerance, remained statistically unchanged across treatments. The level of oxidative stress generated were insufficient to trigger a compensatory accumulation of these accessory pigments, suggesting that the treatments did not result in detectable photooxidative stress on the plants. This stability indicates that neither the fungicide nor pathogen under the experimental condition exposure elicited substantial alterations in photoprotective pigment metabolism possibly due to prior protection by other defense mechanisms.

**Figure 7:**
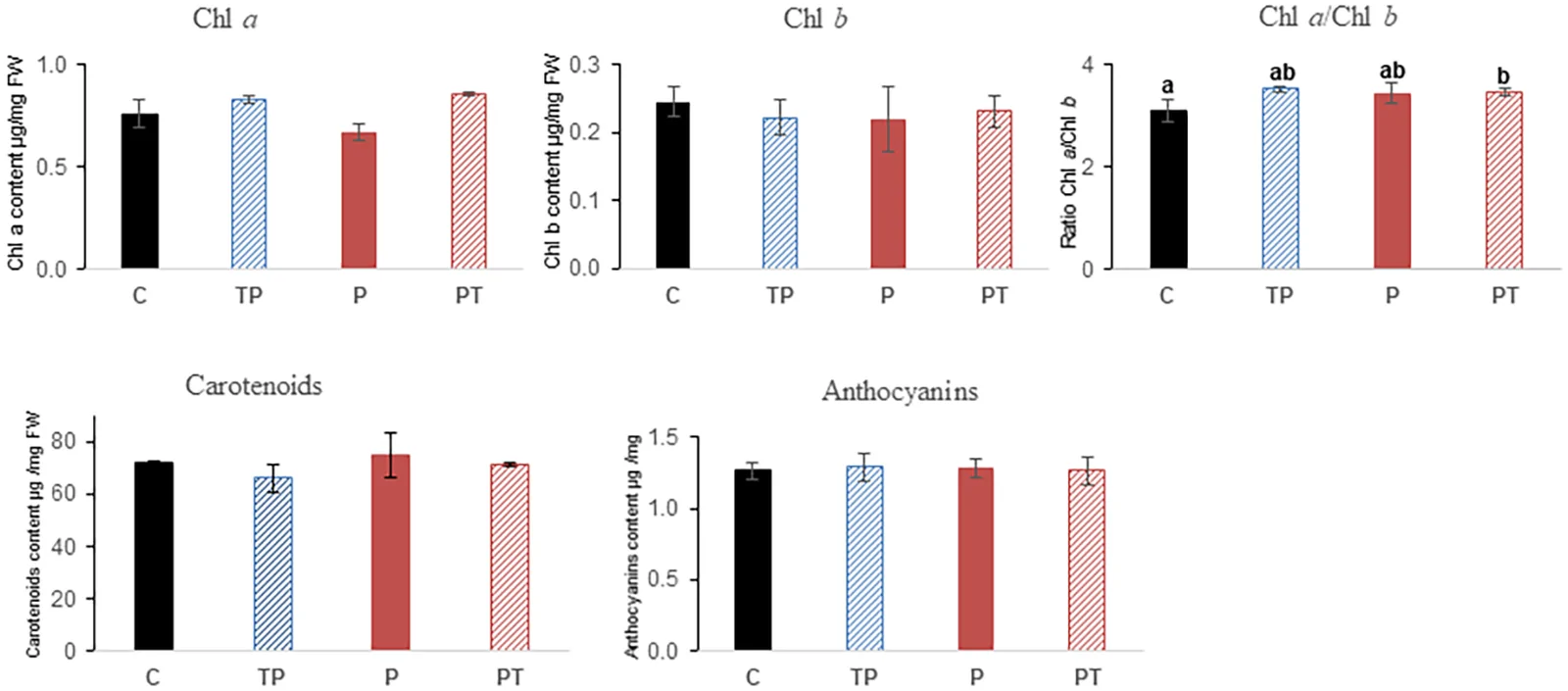
Pigments contents of chlorophyll *a* (Chl *a*), chlorophyll *b* (Chl *b*), the Chl *a*/Chl *b* ratio, carotenoids, and anthocyanins in *P. sativum* plants after 9 days (Day 9) of treatments. C, under control conditions; P, inoculated with *A. pisi*; TP, treated with tebuconazole 24h (T) and inoculated with *A. pisi* (TP); PT, infected plants (P) and sprayed with tebuconazole. Different letters in the bars indicate significant differences (p ≤ 0.05).

### 3.4 Photosynthetic protein content as markers of stress response

cFBP1, TRX *f*, PsaC, D1/PsbA, and LHCII were selected as representative candidate molecular markers associated with chloroplast carbon metabolism, redox regulation, photosynthetic performance and the functionality of photosystems I and II. Western blot analysis of total proteins extracted from pea leaves at D9 using antibodies against the above mentioned key photosynthetic proteins revealed distinct treatment-dependent changes in the abundance of several components of the photosynthetic machinery (**Figure 8**). Quantification of immunoreactive bands showed that cFBP1, a key enzyme of the Calvin-Benson cycle, was reduced by at least 50% in P infected plants, indicating impaired photosynthetic carbon metabolism. Although the decrease was not statistically significant in TP treated plants, cFBP1 abundance seems to remain below control levels. In contrast, PT treated plants exhibited higher cFBP1 abundance (**Figure 8A**). These contrasting responses suggest that pre- versus post-infection fungicide application may differentially modulate carbon metabolism in *A. pisi*-infected pea plants. The redox regulator TRX *f*, which specifically activates cFBP1 through reductive modulation, also showed a reduction of more than 50% in response to P infection on D9. Notably, both pre- and post-fungicide treatments partially restored TRX *f* abundance, approaching those observed in C plants. These may reflect a partial recovery of chloroplast redox homeostasis and carbon assimilation under both combined treatments.

**Figure 8:**
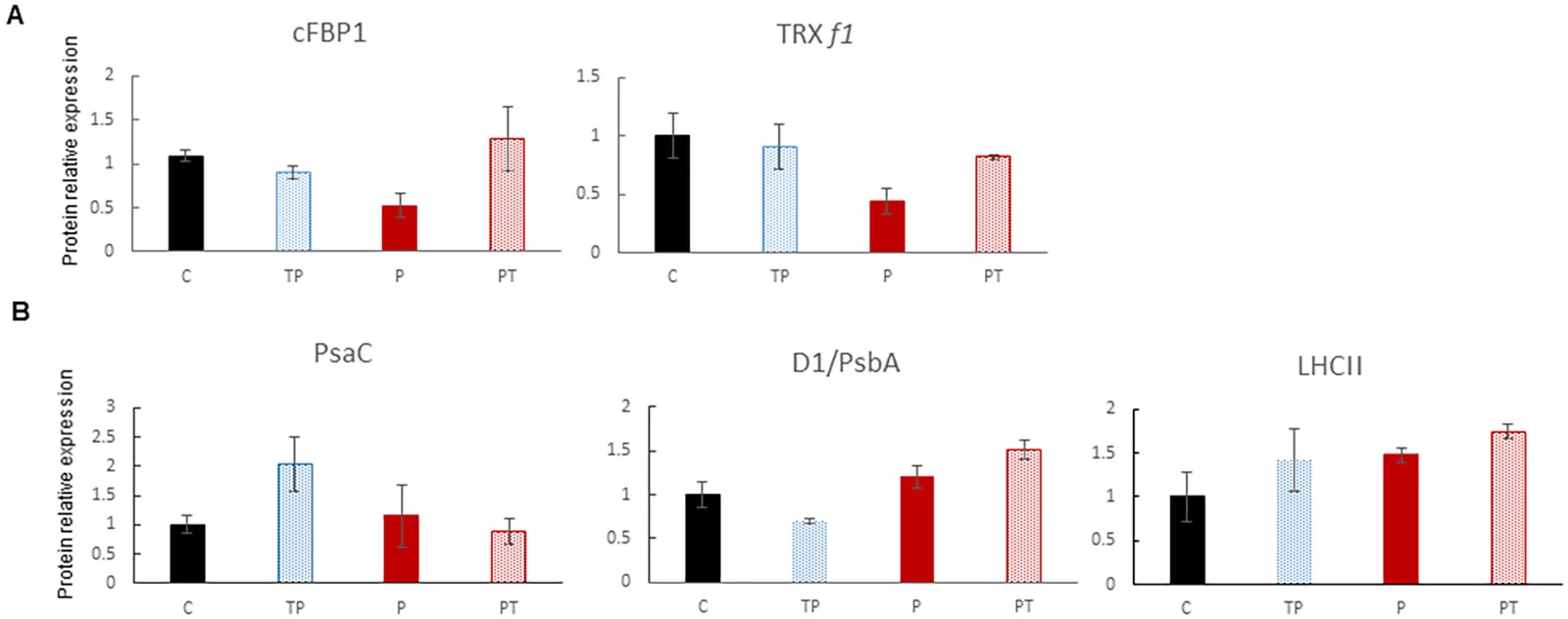
Western blot analysis of **(A)** cFBP1 and TRX *f1*, and **(B)** Psa C, D1 (PsbA) and LHCII proteins in leaves of *P. sativum* plants after nine days (D 9) of different treatments. C, under control conditions; P, inoculated with *A. pisi*; TP, treated with tebuconazole 24h (T) and inoculated with *A. pisi* (TP); PT, infected plants 24h (P) and sprayed with tebuconazole.

Analysis of proteins associated with photosystems I and II revealed distinct responses to the different treatments (**Figure 8B**). PSI subunit PsaC increased approximately twofold in relation to the C plants under TP treatment, whereas no substantial changes were observed in P and PT treated plants. In contrast, plants subjected to P and PT treatments displayed a pronounced increase in PSII proteins, D1/PsbA and LHCII. D1/PsbA protein abundance decreased slightly in TP plants, but increased by approximately 20% and 50% in P and PT treated plants, respectively. Additionally, a significant increase of approximately 50% in LHCII abundance was detected in P and PT treated pea plants. The enhanced accumulation of D1/PsbA and LHCII may represent compensatory remodelling mechanisms within the photosynthetic apparatus, as well as protective responses to pathogen infection and fungicide application potentially contributing to the stabilization of PSII function and optimisation of light harvesting, with possible indirect benefits for PSI.

### 3.5 Principal component analysis of pigment and anthocyanin content, photosynthetic proteins and chlorophyll fluorescence parameters

Principal component analysis (PCA) was used to integrate chlorophyll fluorescence parameters, pigment content and the abundance of selected photosynthetic proteins in pea plants subjected to the different treatments, enabling the identification of coordinated response patterns among variables (Jolliffe and Cadima, 2016; Pérez-Bueno et al., 2019). As shown in **Figure 9**, PC1 and PC2 together explained 95.1% of the total variance, accounting for 62.6% and 32.5%, respectively, indicating that the PCA captured most of the variability in the dataset.

**Figure 9:**
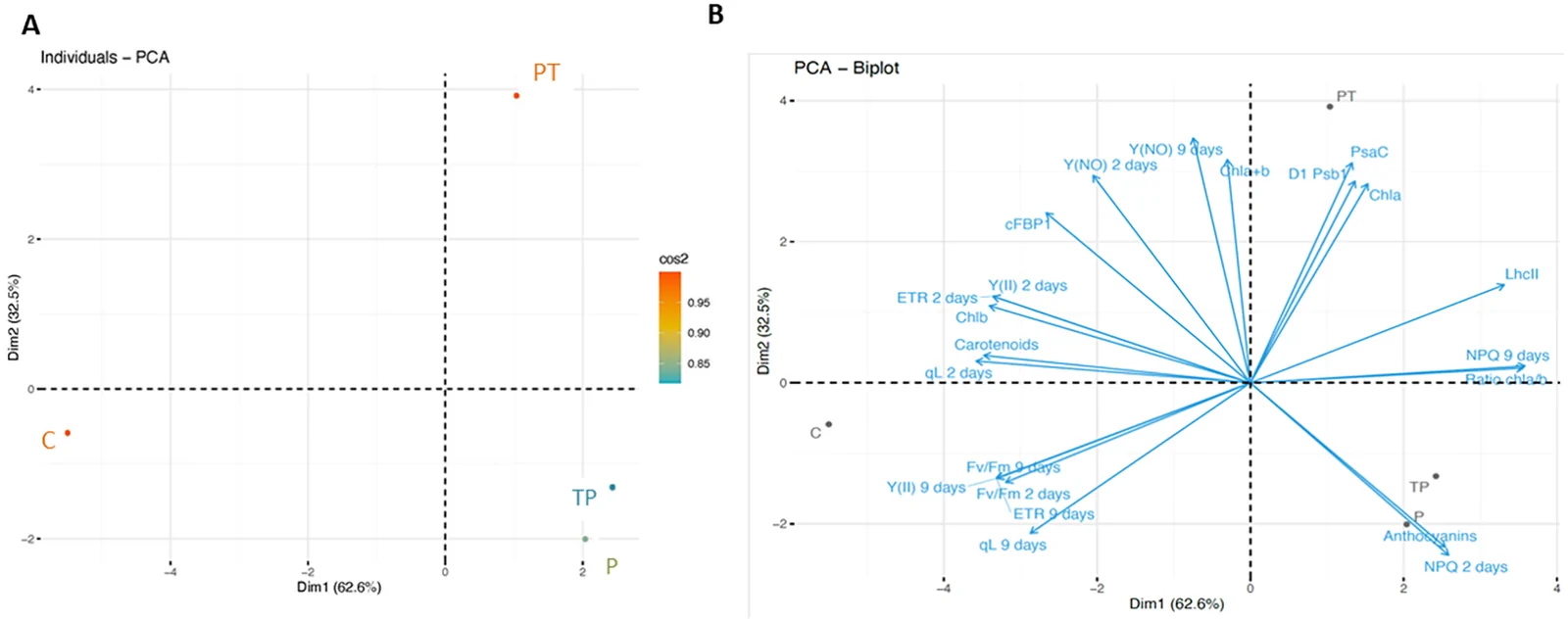
Principal Component Analysis (PCA) integrating chlorophyll fluorescence parameters, pigment content and photosynthetic protein abundance in *Pisum sativum* plants subjected to the different treatments. Plants were maintained under control conditions (C), inoculated with *A. pisi* (P), treated with tebuconazole before pathogen inoculation (TP), or treated with tebuconazole after pathogen infection (PT). **(A)** PCA score plot showing the distribution of samples according to treatment. **(B)** PCA loading plot showing the contribution of chlorophyll fluorescence parameters, pigment-related traits and photosynthetic protein markers to the principal components. Vector direction indicates the association of each variable with the principal components, whereas vector length reflects its relative contribution to the multivariate distribution.

The PCA score plot showed a clear separation among treatments (**Figure 9A**). Control plants (C) were separated from treated plants, whereas infected plants (P) and plants treated with tebuconazole before pathogen inoculation (TP) showed a closer distribution. By contrast, plants treated with tebuconazole after pathogen infection (PT) occupied a distinct position. The loading plot showed that treatment separation was mainly driven by variables related to photosynthetic performance, pigment composition and protein abundance (**Figure 9B**). Among them, ETR, Y(II), qL, chlorophyll-related parameters and carotenoids contributed to the discrimination between control and treated plants. Protein markers contributed to treatment separation by representing changes in PSI (PsaC), PSII (D1/PsbA), light harvesting (LHCII), carbon metabolism (cFBP1) and chloroplast redox regulation (TRX *f*). Variables related to photoprotective and stress-associated responses, NPQ, Y(NPQ) and Y(NO) provided complementary information to the photochemical values, contributing to the separation of treatments through differences in the balance between photochemical activity and non-photochemical energy dissipation.

The PCA showed that P and TP plants had closely related multifactorial profiles, suggesting that tebuconazole pre-treatment had a limited effect on the subsequent overall response induced by the pathogen. In contrast, PT plants were distinct from both the P/TP cluster and the control group. This indicates that tebuconazole application after infection generated a different physiological response to the other treatments.

## 4 Discussion

Early detection of biotic and abiotic stresses in crops, together with the identification of appropriate management strategies, is essential for maintaining agricultural productivity and sustainability. In this context, the present study aimed to identify reliable physiological and biochemical markers of stress and recovery in agronomically relevant plant species. Particular attention was given to the effects of fungicide treatments and their potential role in mitigating pathogen-induced damage and promoting the restoration of photosynthetic function. To investigate the combined effects of biotic (pathogen) and abiotic (fungicide) stressors, a scenario frequently encountered in agricultural systems, we performed *in vitro* experiments using *A. thaliana* for preliminary adjustments and soil-grown conditions with *P. sativum* for pathogen-fungicide treatments. Plants were exposed to two factors: the pathogen *A. pisi* (P) and the fungicide tebuconazole (T). Although the individual effects of fungicides on plant physiological, biochemical, and metabolic processes have been extensively reported, studies addressing their interaction with pathogen infection remain limited. Since tebuconazole is a well-established fungicide, therefore, we evaluated the combined impact of these factors by analysing chlorophyll fluorescence parameters, pigment composition, and photosynthesis-related protein profiles. To ensure a robust and reliable analysis, we conducted a principal component analysis (PCA) integrating all collected data, thereby contributing the development of crop resistance strategies. This approach enabled the identification of pathogen stress-induced alterations and fungicide-mediated responses, providing insights into the mechanisms underlying plant adaptation and recovery under combined biotic and abiotic stress conditions.

### 4.1 Physiological effects on *A. thaliana* plants grown in pesticides

*A. thaliana* plants grown in a sucrose-free medium exhibited a significant reduction in rosette size and biomass, accompanied by a marked increase in chlorophyll, carotenoid, and anthocyanin contents when exposed to a broad range of tebuconazole concentrations. These stress-induced responses to fungicide exposure are likely associated with alterations in photosynthetic performance. In contrast, plants grown in a sucrose-supplemented medium displayed reduced sensitivity to tebuconazole, maintaining biomass and pigment levels comparable to controls except at the highest T concentration tested. Sucrose may mitigate pesticide-induced toxicity, potentially through the activation of reactive oxygen species (ROS) scavenging and detoxification pathways, as previously reported by Ramel et al. (2007). Collectively, these findings suggest that sucrose plays a protective role against fungicide-induced phytotoxicity, particularly at elevated tebuconazole concentrations. Interestingly, the maintenance of plant biomass despite the reduced plant size may be associated with the growth-regulating effects of triazole fungicides rather than their antifungal activity. Triazoles are known to interfere with gibberellin biosynthesis and hormonal homeostasis through the inhibition of cytochrome P450-dependent enzymes. This can lead to altered plant architecture and a redistribution of assimilates toward biomass accumulation by modifying carbon metabolism and reducing growth-associated carbon consumption, effects that may be further amplified under nutrient restricted conditions (Horn et al., 2013; Rogach et al., 2020b). Overall, our findings demonstrate that fungicide exposure significantly affects plant growth, pigment composition, and photosynthetic performance beyond specific concentration thresholds. Growth inhibition induced by increasing tebuconazole concentrations was markedly exacerbated under nutrient-limited conditions in *A. thaliana*, indicating that nutritional status strongly influences fungicide sensitivity. These observations highlight the importance of considering nutritional context when evaluating fungicide effects on plant growth and stress resilience. Despite the clear dose-dependent responses observed in this study, plant reactions to pesticide exposure vary considerably among species and environmental conditions. Comparative studies in agronomically important crops have reported divergent alterations in pigment composition following exposure to different pesticide formulations (Rogach et al., 2020a; Zhang et al., 2010; Moreyra et al., 2019; Serra et al., 2015; Mohsin et al., 2020). For example, investigations in *Vicia faba* and *Vigna unguiculata* have shown that species-specific physiological traits and experimental conditions strongly influence the biochemical outcomes of pesticide application by reducing Chl *a* and Chl *b* (Singh, 2021; Mishra et al., 2008). In addition, Yıldırım et al. (2025) reported that pigment content in *Lolium perenne* is influenced not only by fertiliser type but also by stress-related metabolic responses, highlighting the complex interactions between external inputs and plant physiology. These studies underscore the need for species-specific and context-dependent assessments when evaluating the biochemical and physiological consequences of pesticide exposure. Tebuconazole application induced a decrease in cFBP1 level particularly at high concentrations, with more pronounced effects under sucrose-deficient conditions that likely contributed to impaired photosynthetic carbon assimilation and the activity of specific key enzymes, as it has been described by Petit et al. (2012). However, the relatively moderate decline in cFBP1 abundance suggests the activation of compensatory mechanisms that partially maintain chloroplast function and carbon metabolism under fungicide-induced stress. These findings emphasise the importance of integrating optimal nutritional management into pesticide-use strategies, which is critical for enhancing plant tolerance and improving the effectiveness of endogenous protective mechanisms to promote sustainable crop production.

### 4.2 Linking photosynthetic parameters to the management of *P. sativum* infected with *A. pisi*

Pea is a leguminous crop of increasing agronomic relevance as alternative protein sources. In addition to its nutritional importance, pea cultivation contributes to soil fertility through biological nitrogen fixation, thereby reducing the need for synthetic fertilisers. Nevertheless, under actual field conditions, optimising crop management practices to cope with both biotic and abiotic stresses remains essential. Among biotic constraints, ascochyta blight represents a major threat, leading to significant yield losses. Various fungicides have been reported to be effective against *A*. *pisi*. For instance, Aguilar et al. (2021) demonstrated that *in vitro* the fungicide tebuconazole, alone or in combination, completely inhibited mycelial growth of *A. pisi* as well as other fungal pathogens such as *Colletotrichum lindemuthianum* and *C. truncatum*. A critical question in crop protection concerns whether fungicides should be applied preventively or curatively. To address this issue, in this study we evaluated the physiological responses of pea plants subjected to biotic stress (*A. pisi* infection), abiotic stress (tebuconazole treatment), and their sequential combinations (PT or TP). Our results highlight that both treatment and application timing are key determinants of physiological recovery, crop resilience, and the effectiveness of disease management strategies. In this context, approaches using Imaging PAM (Pulse-amplitude modulation) fluorometry, has emerged as a robust, non-invasive, rapid and sensitive technique for assessing PSII functionality under a wide range of environmental and physiological conditions (White and Critchley, 1999; Schreiber, 2004; Demmig-Adams et al., 2014; Huang et al., 2021). This technique enables the early detection of physiological alterations induced by pathogen infection and provides high spatial and temporal resolution for mapping stress responses across tissues and developmental stages (Pérez-Bueno et al., 2019). In our study, tebuconazole-treated pea plants exhibited moderate reductions in F_v_/F_m_ compared with controls, but impairment was less pronounced than pathogen-infected plants at both D1 and D9, indicating that the fungicide itself imposed a mild physiological cost while simultaneously reducing the severity of pathogen-induced damage. Several studies have shown that triazole fungicides can modify chlorophyll fluorescence parameters, growth patterns, and antioxidant responses, even in the absence of pathogen pressure reflecting physiological adjustments (Petit et al., 2012). This balance between phytotoxicity and disease control is consistent with previous observations that triazole fungicides can act both as stressors and as protectants depending on application rate, timing, crop species, and environmental conditions. Decreases in F_v_/F_m_ are typically associated with photoinhibition or structural damage to the photosynthetic apparatus of PSII (Berova and Zlatev, 2000; Maxwell and Johnson, 2000; Dias, 2012; Zhang et al., 2015).

The decline of both ETR and Y(II) by *A. pisi* infection and partially reversed after tebuconazole application (PT) at the early time point (D2), suggests a transient recovery of photosynthetic activity. Fungicide application likely reduces pathogen pressure and associated oxidative damage, enabling partial restoration of PSII function. These observations highlight the dynamic nature of PSII responses to combined stress. However, the transient nature of this recovery suggests that repeated fungicide applications may be required to maintain protection under sustained infection.

NPQ plays a central role in preventing photodamage of PSII by dissipating excess excitation energy as heat (Lambrev et al., 2012; Murchie and Lawson, 2013). Infected plants exhibited up to a 50% increase in NPQ relative to controls, indicating sustained stress and a continuous requirement for photoprotection. The early enhancement of NPQ, even in the absence of visible symptoms, further supports its utility as a sensitive and initial indicator of physiological disturbance during pathogen infection (Berger et al., 2007a, b). In contrast, the slight reduction of NPQ in tebuconazole-treated plants suggests effective mitigation of pathogen-induced stress and partial alleviation of the requirement for photoprotective energy dissipation. A deeper analysis of photoprotective mechanisms shows that the increase in Y(NO) observed in infected plants may reflect a loss of regulatory capacity within PSII and enhanced non-regulated energy dissipation, a response previously reported during pathogen infection in wheat affected by *Puccinia triticina* (Bürling et al., 2011). In our study, the integration of additional fluorescence parameters such as Y(NPQ) and Y(NO), further supports these findings providing a comprehensive view of PSII energy partitioning and strengthens the conclusion that pathogen infection shifts this balance toward different photoprotective mechanisms, whereas fungicide treatment partially restores photochemical efficiency in a regulated non-photochemical dissipation.

Photosynthetic pigment analysis further complemented the fluorescence data. On D9, only chlorophyll *a*/*b* ratio of P and TP treated pea plants varied relative to the control, indicating that both pathogen infection and fungicide application affected photosynthetic functionality followed by an adjustment in pigment composition. An increased ratio suggests modifications in light-harvesting complexes and potential acclimation to stress conditions aimed at optimising energy capture (Evans, 1989; Tanaka and Tanaka, 2006). An elevated Chl *a*/Chl *b* ratio is typically associated with a reduction in the size of the LHCII antenna and may reflect an acclimatory response aimed at optimising efficient energy transfer and photosynthetic performance under stress or altered physiological conditions. Such a response supports the notion that chlorophyll fluorescence parameters are often more sensitive indicators of early physiological stress than pigment content alone, allowing the detection of functional disturbances prior to the appearance of structural damage to the photosynthetic apparatus. Nevertheless, pigment content serves as a valuable marker for accurately assessing physiological status and the effectiveness of defense mechanisms. In addition, carotenoid and anthocyanin levels stability, suggests an effective activation of antioxidant and protective mechanisms that maintained pigment homeostasis (Demmig-Adams and Adams, 1996; Gould, 2004).

### 4.3 Potential use of photosynthetic proteins involved in carbon metabolism and electron transport as markers of plant stress responses

Proteins associated with the photosynthetic electron transport chain and the Calvin–Benson cycle are frequently modulated in response to both biotic and abiotic stresses, reflecting alterations in energy transduction, redox homeostasis, and carbon assimilation (Foyer et al., 2009; Tang et al., 2011). In *A. thaliana* and *P. sativum*, stress-induced changes in key photosynthetic protein and core components of photosystems I and II, have been widely reported, supporting their use as sensitive molecular markers of plant physiological status under adverse conditions. cFBP1 suppression in *Arabidopsis* severely affects plants growth and alters carbon partitioning, particularly the sucrose-to-starch ratio leading to the photosynthetic impairment (Rojas-González et al., 2015). In our preliminary experiments *Arabidopsis* cFBP1 abundance decreased following fungicide treatment, an effect aggravated under sucrose-deficient conditions. In *P. sativum* plants infected with *A. pisi* the abundance of both cFBP1 and TRX *f* decreased by approximately 50%. Notably, both pre- and post-infection applications of tebuconazole treatments partially mitigated this decline, suggesting that fungicide application might preserve carbon assimilation capacity and redox-regulated metabolic processes under pathogen stress. Consistent with this interpretation, enhanced TRX dependent regulation systems have been associated with improved stress tolerance, greater photosynthetic stability, and increased resilience to environmental challenges (Thormählen et al., 2015).

Pathogen-induced reductions in photosynthetic activity are often associated with extensive reprogramming of carbohydrate metabolism and source-sink relationships (Kanwar and Jha, 2019). Beyond their role as carbon resources, soluble sugars act as signalling molecules that coordinate redox homeostasis, hormone signalling, and defense-related metabolic pathways (Kanwar and Jha, 2019). Future research integrating carbohydrate profiling with photosynthetic and proteomic analyses may reveal whether the restoration of photosynthetic function by fungicide treatment is linked to the recovery of carbon metabolism, source-sink dynamics, and sugar-mediated defense signalling. Pathogen infection is known to induce oxidative stress, damaging key PSII components such as the D1/PsbA and LHCII, thereby impairing electron transport (Berger et al., 2007a, b; Gururani et al., 2015). In our study, proteins associated with the photosynthetic electron transport chain, PsaC (PSI) increased under TP treatment, while the D1/PsbA protein (PSII) decreased under the same treatment but increased under P and PT conditions; finally, LHCII accumulated in all treatments relative to the control. This increase, mainly of PSII components, likely reflects a compensatory response aimed at preserving electron transport capacity toward PSI core and maintaining photosynthetic performance under stress conditions. Such adjustments are consistent with the chlorophyll fluorescence data, particularly the partial recovery observed in ETR and Y(II), and may indicate a reorganisation of the photosynthetic apparatus in response to altered energy demands and stress-associated damage. Enhanced turnover and accumulation of D1/PsbA, PSI components, and antenna proteins are well-documented mechanisms involved in the repair of photodamaged photosystems and the optimisation of light energy utilisation under oxidative stress (Aro et al., 1993; Tikkanen and Aro, 2014). Variations in the abundance of proteins such as TRX *f*, cFBP1, PsaC, D1/PsbA, and LHCII, reflect coordinated adjustments in redox homeostasis, carbon fixation, and photosynthetic electron transport. Together, these results support the value of photosynthesis-related proteins as sensitive molecular markers of plant stress responses triggered by fungicide treatments or pathogen infections.

### 4.4 Multivariate integration of physiological and molecular responses reveals treatment-specific photosynthetic strategies

Multivariate approaches such as PCA are widely employed in plant physiology to identify coordinated responses to biotic and chemical stress and reduce dimensionality, thereby facilitating the identification of key traits underlying stress adaptation (Pérez-Bueno et al., 2019). Integrating chlorophyll fluorescence imaging, physiological parameters and photosynthetic protein molecular markers has offered a powerful approach for characterising plant stress responses, improving the early detection of biological dysfunction, and supporting the development of more effective and sustainable crop management strategies. The PCA results indicate mainly that tebuconazole partially alleviated the impact of infection on *P. sativum*, shifting treated plants toward the resilient phenotype and consistent with a curative rather than a preventive role. In another hand, the PCA provide some evidence that the timing of fungicide application relative to pathogen infection is an important determinant of plant physiological responses. From an applied perspective, this finding may highlight the value of optimising fungicide treatment strategies not only for disease control but also for preserving photosynthetic performance, metabolic stability, yield and crop resilience under pathogen pressure.

## 5 Conclusions

This study provides a comprehensive assessment of the physiological, biochemical, and molecular responses of *P. sativum* to *A. pisi* infection and tebuconazole treatment. By integrating chlorophyll fluorescence imaging, pigment profiling, protein analysis, and multivariate statistics, we showed that pathogen infection and fungicide application induced distinct but coordinated responses in the photosynthetic apparatus.

Among the evaluated parameters, chlorophyll fluorescence traits, pigment composition and photosynthesis-related proteins, including cFBP1, TRX *f*, PsaC, D1/PsbA, and LHCII, may serve as sensitive indicators of stress and recovery. Multivariate analysis further identified the most informative markers for distinguishing treatment effects and assessing plant physiological status.

Overall, this study demonstrates that the integration of physiological, biochemical, and molecular markers provides a robust framework for evaluating plant resilience under combined stress conditions. Future research should investigate the long-term and dose-dependent effects of fungicide applications, across different crop variety and incorporate transcriptomic and metabolomic approaches to further elucidate the mechanisms underlying plant stress adaptation.

## Data Availability

The original contributions presented in the study are included in the article/**Supplementary Material**, further inquiries can be directed to the corresponding author/s.

## Supplementary material

Supplementary Figure 1 TRX *f* protein relative content determined by western blot analysis in 20 days old *Pisum sativum* plant leaves spread with water (Control) or with *Ascochyta pisi* diluted at 1/10,000, 1/1,000, 1/100, 1/10 and undiluted. This is the result of three replicas and normalized with actin.

Supplementary Table 1 PSII open reaction centers, qL, values at D1, D2 and D9 measured with Imaging Pam fluorescence.
