# Supplementary material for: Photosynthetic markers reveal recovery of Pisum sativum from pathogen infection following fungicide treatment
Source: Front Plant Sci. 2026 Oct 30;17:2005488. doi: 10.3389/fpls.2026.2005488 (PMC13759756; doi:10.3389/fpls.2026.2005488)
Supplement: Supplementary file 1 [file DataSheet1.pdf]

Supplemental Figure 1

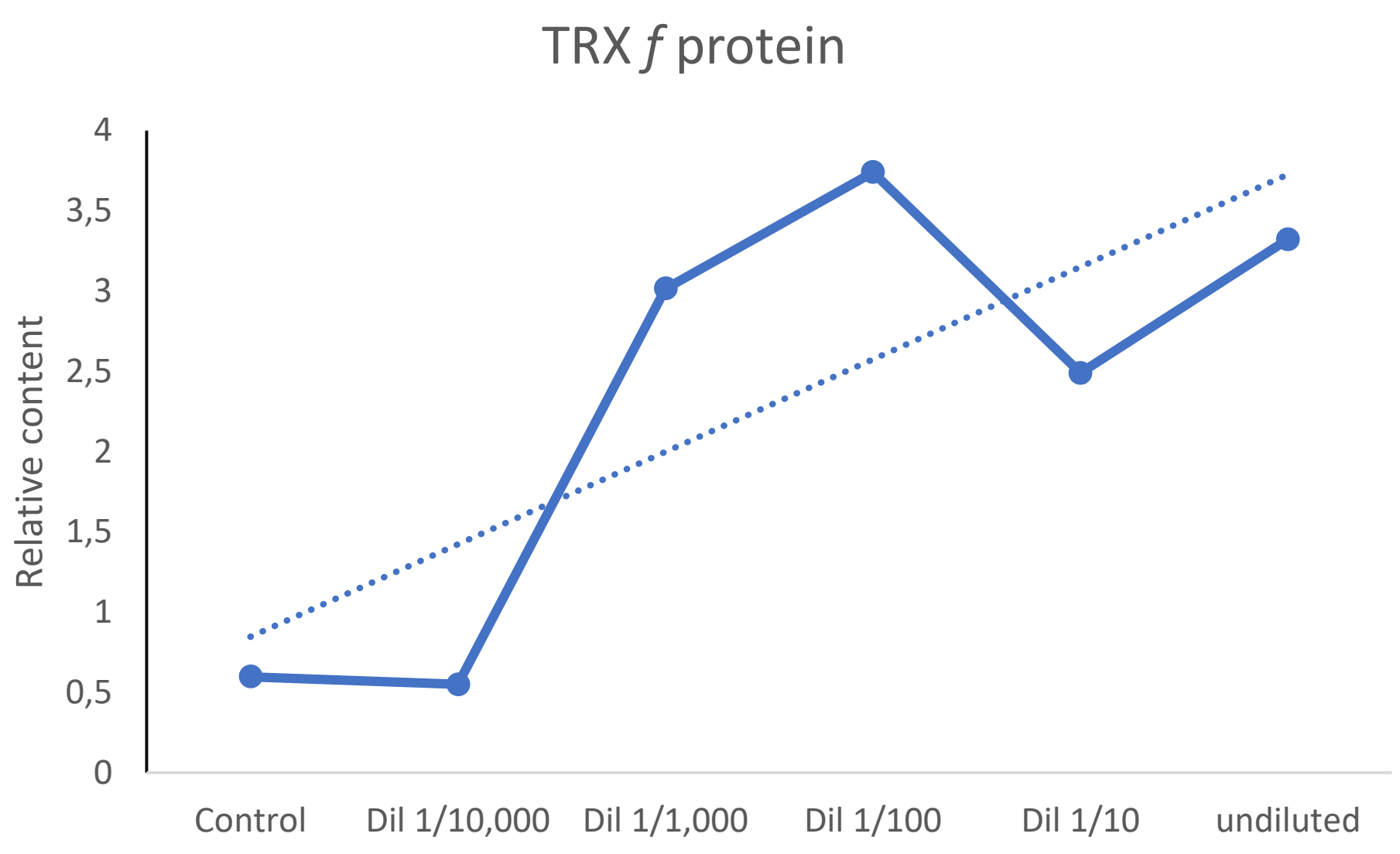

Supplemental Figure 1 TRX *f* protein relative content determined by western blot analysis in 20 days old *Pisum sativum* plant leaves spread with water (Control) or with *Ascochyta pisi* diluted at 1/10,000; 1/1,000; 1/100; 1/10 and undiluted. This is the result of three replica and normalized with actin.
