# Supplementary material for: Photosynthetic markers reveal recovery of Pisum sativum from pathogen infection following fungicide treatment
Source: Front Plant Sci. 2026 Oct 30;17:2005488. doi: 10.3389/fpls.2026.2005488 (PMC13759756; doi:10.3389/fpls.2026.2005488)
Supplement: Supplementary file 2 [file DataSheet2.pdf]

Supplemental Table 1. PSII open reaction centers, qL, values at D1, D2 and D9 measured with Imaging Pam fluorescence

	Day 1			Day 2				Day 9			
**PAR/Treatm**	**C**	**T**	**P**	**C**	**TP**	**PT**	**P**	**C**	**TP**	**PT**	**P**
**0**	1	1	1	1	1	1,000	1,000	1	1	1	1
**1**	0,888	0,884	0,997	0,903	0,907	0,906	0,910	0,879	0,883	0,889	0,882
**11**	0,559a	0,606ab	0,633b	0,746	0,752	0,700	0,726	0,661	0,568	0,543	0,606
**21**	0,479	0,509	0,526	0,613	0,622	0,547	0,607	0,515	0,551	0,490	0,565
**36**	0,449	0,509	0,526	0,553	0,595	0,540	0,600	0,521	0,523	0,460	0,535
**56**	0,500	0,545	0,546	0,577	0,614	0,573	0,616	0,501	0,475	0,415	0,489
**111**	0,469	0,477	0,473	0,566	0,561	0,525	0,571	0,376	0,327	0,286	0,339
**146**	0,480	0,481	0,471	0,559	0,540	0,513	0,553	0,364	0,308	0,261	0,314
**231**	0,428	0,396	0,381	0,492	0,433	0,431	0,455	0,285	0,226	0,185	0,226
**336**	0,358	0,341	0,314	0,418	0,361	0,358	0,362	0,227	0,177	0,137	0,175
**396**	0,354	0,327	0,302	0,406	0,338	0,354	0,337	0,211	0,160	0,125	0,160
**461**	0,343	0,316	0,301	0,394	0,324	0,350	0,314	0,194	0,155	0,123	0,160
**531**	0,334	0,305	0,294	0,373	0,321	0,331	0,285	0,189	0,154	0,131	0,169
**611**	0,315	0,288	0,278	0,336	0,285	0,306	0,259	0,183	0,149	0,131	0,157
**801**	0,247	0,232	0,223	0,274b	0,233ab	0,243ab	0,196a	0,142	0,126	0,118	0,129
**1076**	0,121	0,179	0,165	0,192b	0,189b	0,182ab	0,146a	0,114	0,099	0,088	0,100
**1251**	0,095	0,049	0,071	0,158	0,164	0,152	0,141	0,099	0,084	0,078	0,092

Different letters for the same light intensity and different treatment indicate significant differences ($p \leq 0.05$) ($n=5$)
